# Respiratory Rate Monitoring via a Fibre Bragg Grating-Embedded Respirator Mask with a Wearable Miniature Interrogator

**DOI:** 10.3390/s24237476

**Published:** 2024-11-23

**Authors:** Nat Limweshasin, Itzel Avila Castro, Serhiy Korposh, Stephen P. Morgan, Barrie R. Hayes-Gill, Mark A. Faghy, Ricardo Correia

**Affiliations:** 1 Optics and Photonics Research Group, Faculty of Engineering, University of Nottingham, Nottingham NG7 2RD, UK; nat.limweshasin1@nottingham.ac.uk (N.L.); itzel.castro@nottingham.ac.uk (I.A.C.); s.korposh@nottingham.ac.uk (S.K.); barrie.hayes-gill@nottingham.ac.uk (B.R.H.-G.); ricardo.goncalvescorreia@nottingham.ac.uk (R.C.); 2 Biomedical and Clinical Research Theme, School of Human Sciences, University of Derby, Derby DE22 1GB, UK; m.faghy@derby.ac.uk

**Keywords:** respiration rate, fibre bragg grating, temperature, ambulatory, wearable

## Abstract

A respiration rate (RR) monitoring system was created by integrating a Fibre Bragg Grating (FBG) optical fibre sensor into a respirator mask. The system exploits the sensitivity of an FBG to temperature to identify an individual’s RR by measuring airflow temperature variation near the nostrils and mouth. To monitor the FBG response, a portable, battery-powered, wireless miniature interrogator system was developed to replace a relatively bulky benchtop interrogator used in previous studies. A healthy volunteer study was conducted to evaluate the performance of the developed system (10 healthy volunteers). Volunteers were asked to perform normal breathing whilst simultaneously wearing the system and a reference spirometer for 120 s. Individual breaths are then identified using a peak detection algorithm. The result showed that the number of breaths detected by both devices matched exactly (100%) across all volunteer trials.

## 1. Introduction

Respiration rate (RR), a measure of the number of breaths taken per minute, is a vital sign that plays a crucial role in assessing a person’s overall health. It can provide valuable information for medical diagnosis, patient monitoring, and performance assessment in sports [1,2]. RR monitoring can serve as a means of early detection of respiratory complications. For example, this is important for workers in harsh environments such as factories and mines. Other health monitoring cases include acute exacerbation of COPD [3]; dyspnea of workers due to exposure to volatile organic compounds in automobile manufacturing factories or nuclear waste disposal; and asthma or pneumoconiosis due to inhalation of dust and particles in textile industry workers or miners [4,5]. Presently, RR measurements in these environments typically rely on wearable piezoresistive/piezoelectric strain sensors integrated into a belt to detect breathing [6,7].

Table 1 summarises the principles and limitations of recent advancements in RR monitoring methods. These compute RR by measuring respiration-induced changes in the torso and nostrils, i.e., torso strain, sound, and temperature, whose magnitude varies proportionately with inhalation and exhalation volume [8,9,10,11,12,13,14,15]. Sensing respiration-induced torso strains and imaging body movements are the most popular [9,10,11,13,16,17] as they are used in commercially available RR monitoring devices, i.e., Go Direct Respiration Belt (Vernier, Beaverton, OR, USA) via load cell monitoring of changes due to respiration [18] and BioHarness 3.0 (Medtronic, Minneapolis, MN, USA) utilising a pressure sensor [19] and motion capture systems (e.g., Vicon Motion Systems Ltd., Oxford, UK) [20]. Performance errors in these sensors stem from varying signal quality due to noise from respiration-irrelevant factors that affect the sensing elements, such as camera obstructions (imaging systems) and body motions (pressure sensors). Furthermore, as the load cell and pressure sensors are based on conductive materials, they can be affected by electromagnetic interferences (EMIs) that limit their usage in environments such as factories and coal mines or during magnetic resonance imaging (MRI) [21,22,23,24].

Optical fibre sensor (OFS) technology has also gained in popularity in RR monitoring [25]. Table 2 summarises the characteristics of recently published OFS RR sensors. The capabilities of OFS and conductive/electronic sensors are comparable as both can measure respiration-induced torso strain and breath temperature near the nostril [26,27,28,29,30]. Similar issues are faced, for example, by its conventional conductive sensor counterpart, a strain-based OFS (i.e., Fibre Bragg Grating (FBG)), which can be affected by respiration-irrelevant motions [29,30,31,32]. Furthermore, when performing physically demanding tasks, such as exercises and factory/mining activities, the fibre risks damage. Despite this, OFS technology offers a significant advantage over conductive sensors as it is immune to EMI. This can be useful during MRI [27] or in harsh environments. Conventional sensors require both the electronics and sensing elements to be shielded [21,33].

In this paper, an ambulatory FBG-based RR monitoring system is demonstrated. An FBG optical fibre sensor is embedded in an adjustable respirator mask for comfort as well as to avoid motion artifacts associated with strain-based torso sensors. Exploiting the sensitivity of an FBG to temperature [36], the difference between an individual’s breath and ambient air is compared to instantaneously detect inhalation and exhalation. Previously, we have demonstrated a similar method of RR monitoring using an FBG embedded in oxygen delivery devices [30]. Thus, the novelty of this work is the incorporation of a portable, battery-powered miniature interrogator as a means of data acquisition to facilitate usage during daily life and occupational activities, as well as featuring real-time data display for breath-temperature-based OFS RR monitoring. The performance of the developed system is evaluated against a reference spirometer (Easy On-PC Spirometer, ndd Medizintechnik AG, Zürich, Switzerland) in a volunteer study involving 10 healthy individuals in a controlled environment. The results are then used as the basis of discussion for performance analysis and future plans.

## 2. Materials and Methods

### 2.1. FBG Respirator Mask Design

**Figure 1:**
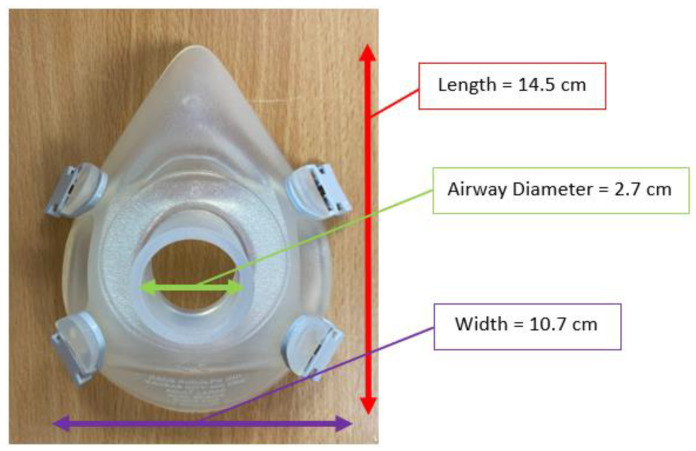
Dimension of employed respirator mask (head strap removed).

**Figure 2:**
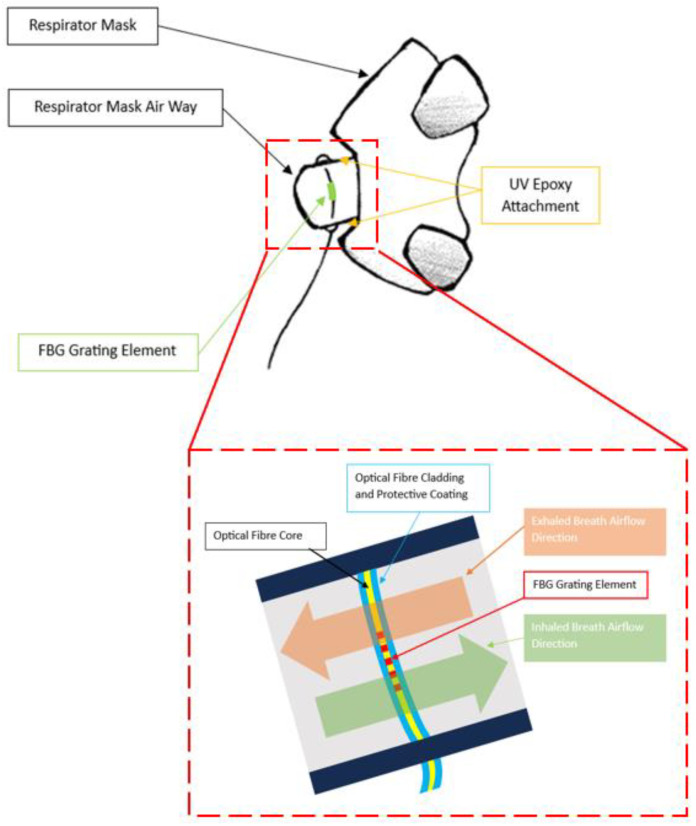
Side view illustration of overall design of respirator mask after FBG placements perpendicular to breath airflow direction; sub-panel shows a magnified sketch (not to scale) at the respirator mask airway and cross-sectional diagram of FBG grating elements along with inhalation and exhalation airflow directions.

The developed system employs a respirator mask with an unobstructed open airway channel (8920 Series, Hans Rudolph Inc., Shawnee, KS, USA) (Figure 1). An optical fibre containing an FBG is placed in the open airway so that the grating element is perpendicular to the opening (Figure 2) to maximise the surface area between the FBG element and breathing airflow. This is achieved by drilling 2 holes (~3 mm diameter) across the airway wall and inserting the FBG, which is then secured such that the fibre is slightly curved to minimise strain and resealed using UV curable epoxy (Norland Optical Adhesive 68, Ultraviolet Curing, Norland Products Inc., Jamesburg, NJ, USA) (Figure 2). The position and orientation of the FBG maximises its sensitivity to airflow temperature variation during breathing.

### 2.2. Sensing Principle

Fibre Bragg Grating (FBG) is a type of optical sensor that is inscribed into a segment of optical fibre. It works by reflecting specific wavelengths of light based on periodic variations in the refractive index within the fibre. These variations create a “grating” that reflects light at a particular wavelength, called the Bragg wavelength ($\lambda_{B})$. When external factors like temperature and strain change, the Bragg wavelength shifts, allowing FBG sensors to monitor these conditions [36]. Additionally, FBG sensors, unless modified, are insensitive to humidity [37], making them suitable for measuring breathing rates through breath-induced air temperature changes. In this work, the detection of inhalation and exhalation relies on the FBG’s sensitivity to temperature variations. This is reflected by variations in the Bragg wavelength, which can be expressed as seen in Equation (1) [34,38,39].

(1) $$∆\lambda_{B}=2(\Lambda \;\frac{\partial n\;}{\partial l}+n\;\frac{\partial \Lambda }{\partial l})\;∆l+2(\Lambda \;\frac{\partial n\;}{\partial T}+\;n\;\frac{\partial \Lambda }{\partial T})\;∆T$$

where $\lambda_{B}$ = FBG Bragg wavelength (m); T = temperature (°C); l = length (m); Λ = grating period (m); n = refractive index [36,37,38].

According to Equation (1), the term “$2(\Lambda \;\frac{\partial n\;}{\partial T}+\;n\;\frac{\partial \Lambda }{\partial T})\;∆T$” represents the sensitivity of the FBG to temperature variation [36]. Upon heating, the length of the optical fibre changes due to thermal expansion. This induces variation in the FBG grating period (Λ), as well as the refractive index (n) [36,39]. This causes $\lambda_{B}$ to increase. The reverse applies when the FBG is cooled. Assuming no strain is applied, the Bragg wavelength shift (∆$\lambda_{B}$) due to temperature can be mathematically written as Equation (2) [36,39].

(2) $$∆\lambda_{B}=\lambda_{B}(\alpha +\zeta )∆T$$

where α = thermal expansion coefficient (approx. 0.55 × 10^−6^ for silica optical fibre; ζ = thermo-optic coefficient (approx. 8.6 × 10^−6^/°C for germanium-doped silica core optical fibre) [36].

### 2.3. Miniature Interrogator Instrumentation

**Figure 3:**
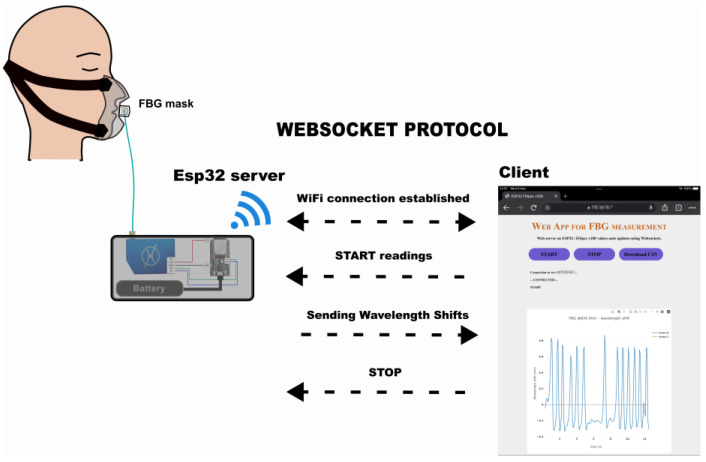
System diagram of the mini-interrogator operation with WebSocket protocol.

In this research, the FBG data are collected using a portable, battery-powered miniature interrogator (FiSpec FBG X100, FiSens GmbH, Braunschweig, Germany) with a precision measurement of 0.1 °C|1 με (10 Hz). This miniature interrogator measures the reflected spectrum of the guided light in the optical fibre with the FBG sensor. This measurement is achieved in a very limited space by integrating a tilted FBG in the core of the optical fibre through inscribing microscopic structures using a proprietary femtosecond laser process. This produces a combination of scattering and diffraction, coupling out the light and breaking it down into wavelengths onto a plane where the image sensor (CCD) collects this light spectrum to be read by the embedded microprocessor of the miniature interrogator. This miniature interrogator was connected to a microcontroller (ESP32-WROOM-32D, Espressif Systems, Shanghai, China) (Figure 3) and powered by a battery. The whole system is referred to collectively as a “mini-interrogator”. The mini-interrogator is placed inside a 3D-printed box (6.5 cm × 15.5 cm × 8.5 cm). Overall, the apparatus weighs 374 g and has a 17.64 h battery lifetime after a full charge (AAA battery, 1100 mAh). Two communication protocols, WebSocket and UART protocol, are utilised. The WebSocket enables communication between the client, the web application that enables the real-time display of the FBG data, and the server of the microcontroller, which processes the FBG data from the interrogator. Meanwhile, the UART protocol establishes the connection between the microcontroller and the interrogator to read the FBG data.

**Figure 4:**
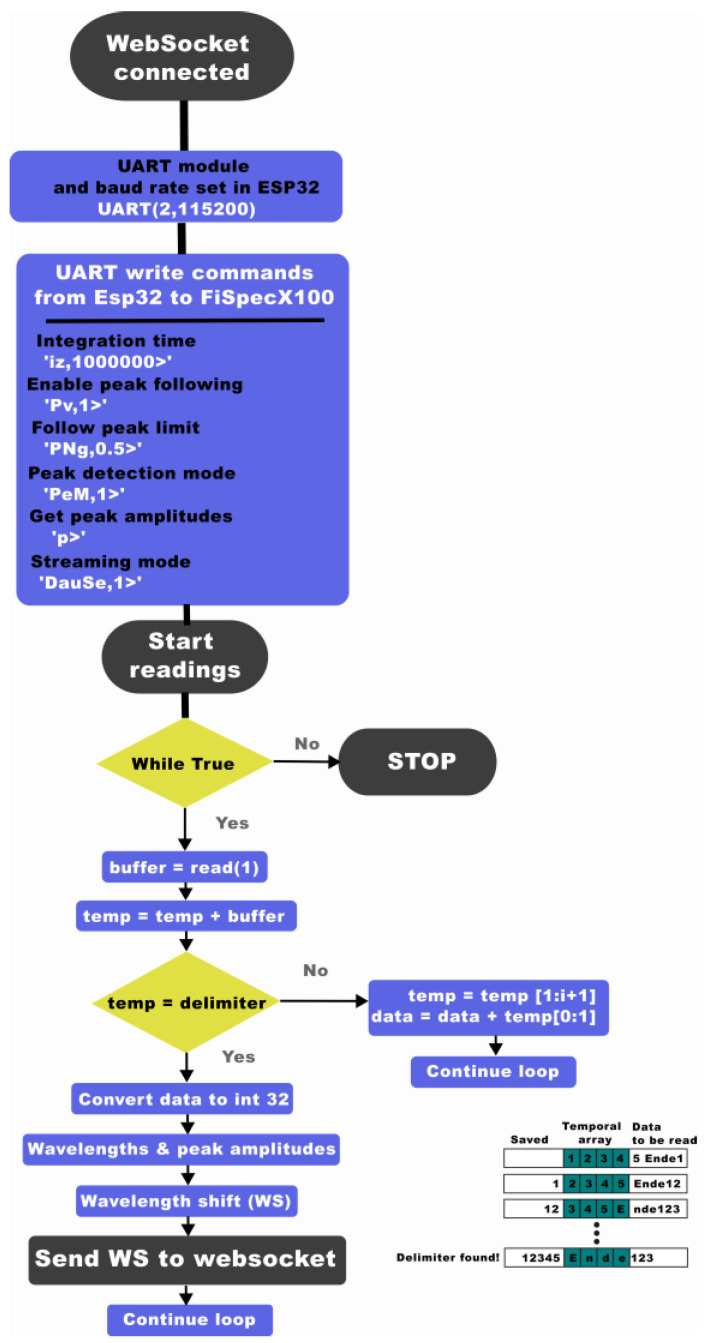
FiSpecX100 instrumentation flow chart, including ESP32, UART protocol, and parsing of FBG data/wavelength shift values.

The operation diagram of the interrogator with the microprocessor is depicted in Figure 3, and the process pipeline to acquire the FBG data from the interrogator using the microprocessor is shown in Figure 4.

The integration time of the mini-interrogator was set to 100 ms. This integration time equals a sampling rate of around 10 Hz, which was the maximum allowed by the external UART connection of FiSpecX100, as it is limited to a baud rate of 115,200. However, it is sufficient to measure breathing rate because its normal value in healthy adults ranges between 0.20 and 0.33 Hz [40]. The follow peak function was enabled with the FiSens interface command “Pv,1>” with a follow peak limit of 50%, and the peak detection mode was set to extreme detection using the 1st derivative. Once the intensity and wavelength values were obtained, the Bragg wavelength at rest is measured, which corresponds to the maximum amplitude peak in the reflected spectrum at rest position. When the temperature changes, the Bragg wavelength will shift, and it is recorded relative to the Bragg wavelength at rest.

Following the calculation of the wavelength shift from the FBG sensor, data are sent through the WebSocket until the client stops it. Additionally, the displayed data can be downloaded in a “.csv” file for further processing.

### 2.4. Volunteer Study

This section explains the setup and protocol to evaluate the “Mask System”, which refers to the FBG respirator mask and the mini-interrogator collectively against the gold standard spirometer. The experiment setup and protocol for Mask System performance evaluation was approved by the University of Nottingham, Faculty of Engineering, Ethics Committee (Ref: 2022-64, approved March 2024, study conducted May–July 2024). Each volunteer gave their informed consent prior to commencing the procedure.

#### 2.4.1. Setup

**Figure 5:**
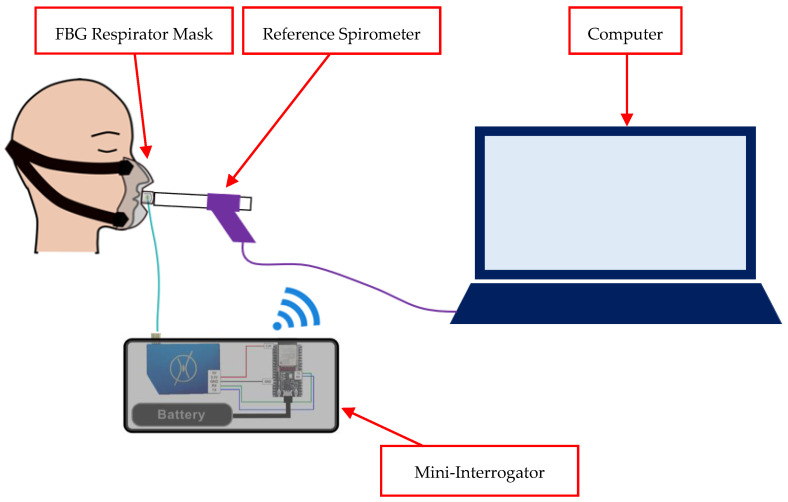
Mask System setup during volunteer study (note: FBG placement on respirator mask is as shown in Figure 2; head strap included in this figure).

**Figure 6:**
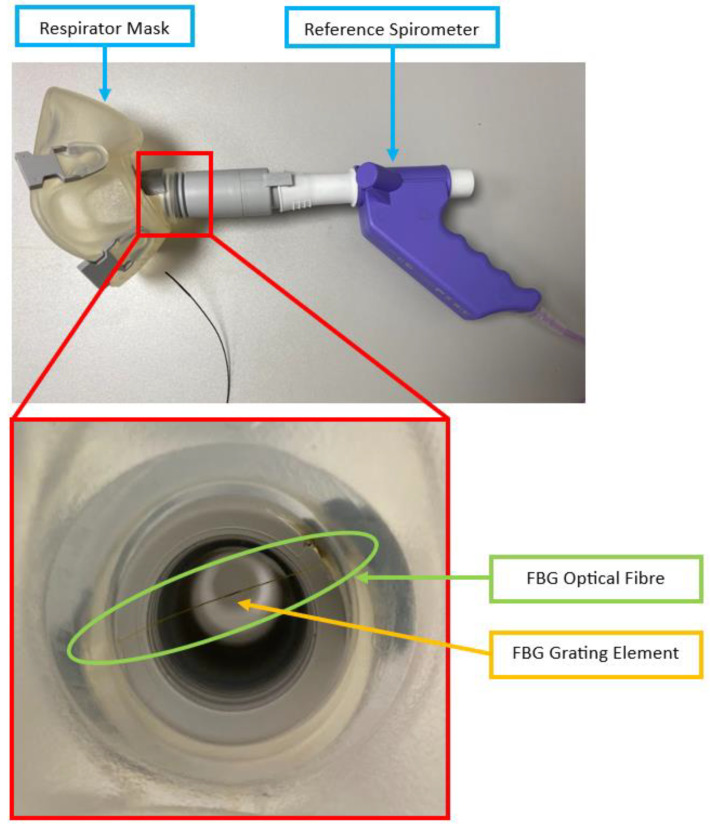
FBG respirator mask and reference spirometer setup during volunteer experiments (sub-panel shows a photo of FBG placement in the respirator mask airway, taken from the inside of the respirator mask after spirometer attachment).

To evaluate the performance of the Mask System, 10 healthy volunteers without known health complications (9 males, 1 female, mean age 28 (2.83 standard deviation)) were recruited. The imbalance between males and females was not intended but was simply dependent on the availability of volunteers. According to previously published research [41], there is no correlation between exhaled breath temperature at rest and gender. This is also the case for body temperature (measured at traditional sites such as axilla), heart rate, height, weight, and blood pressure [41]. Thus, these factors do not affect the measurements in this study. However, it is well documented that during physical activities (such as physical exercises and manual labour), individual breath temperature increases [42]. These will be explored in future work during work-simulated experiments following the FBG and miniature interrogator’s integration into industrial-grade masks, as described in the Discussion section. For confidentiality, each volunteer is given a unique identification number (ID). The procedure involved the volunteers performing natural tidal breathing whilst wearing the Mask System. A spirometer is fitted with the mask for simultaneous reference measurements (Figure 5 and Figure 6). The reference spirometer used in our study (Easy On-PC Spirometer) is an ultrasonic device that measures breathing volume over time. It works by emitting high-frequency sound waves that reflect off the particles in the exhaled and inhaled air, allowing the device to calculate the air volume passing through the sensor. The data acquisition rate of both devices is set to 10 Hz.

The experiment is performed in a closed, air-conditioned lab environment with air conditioning set to 23 °C and maintained throughout the experiment. This means that the ambient temperature is lower than the exhaled breath temperature (approximately 34 °C in healthy individuals [43]). Thus, it is expected that during exhalation, the FBG wavelength data will shift upward as the increase in temperature causes expansion in the FBG grating element, whilst the reverse (downward shift) is expected for inhalation.

#### 2.4.2. Protocol

The breathing protocol includes 5 repetitions of tidal breathing, each lasting 2 min. To align both the Mask System and reference spirometer data, the timer is set to start as soon as the volunteer performs the first exhalation. The volunteers are free to choose their breathing rate and volume, as well as their path of gas exchange (oral or nasal breathing). A 30–60 s break is given between each measurement repetition.

#### 2.4.3. Data Processing and Interpretation

Since it is expected that exhalation and inhalation cause upward and downward FBG wavelength shifts, respectively, it is the opposite of the reference spirometer. Hence, prior to filtering, the Mask System data are inverted. The Mask System data are processed using a moving average filter and MATLAB (R2019b)’s built-in peak detection function “findpeaks” to reduce noise and identify individual breaths, respectively. The moving average filter has a 10-sample window size (equivalent to 1 s at a 10 Hz sampling rate).

In this experiment, only complete breaths are counted. This is defined as an inhalation followed by an exhalation [44]. This is identified in the data from both the Mask System and reference spirometer by locating the peak within the signal that represents peak inhalation. To achieve this, the “minpeakprominence” value of the findpeaks function is set to 7.4 pm for every volunteer Mask System data. This was chosen based on the magnitude of noise associated with the Mask System when placed on a benchtop in the same experiment venue and air conditioning setting as volunteer studies.

For the reference spirometer, breath identification is performed using manual identification. That is, during experimentation, the investigator (main author, N.L.) observes the spirometer waveform simultaneously with the volunteer’s chest wall motion using the same protocol as manual breath counting (rising indicates inhalation and falling indicates exhalation [45,46]). Each complete breath is noted and marked on the peak of the spirometer signal as a reference. The number of complete breaths (peaks) counted from both the Mask System and reference spirometer are recorded and compared.

## 3. Results

**Figure 7:**
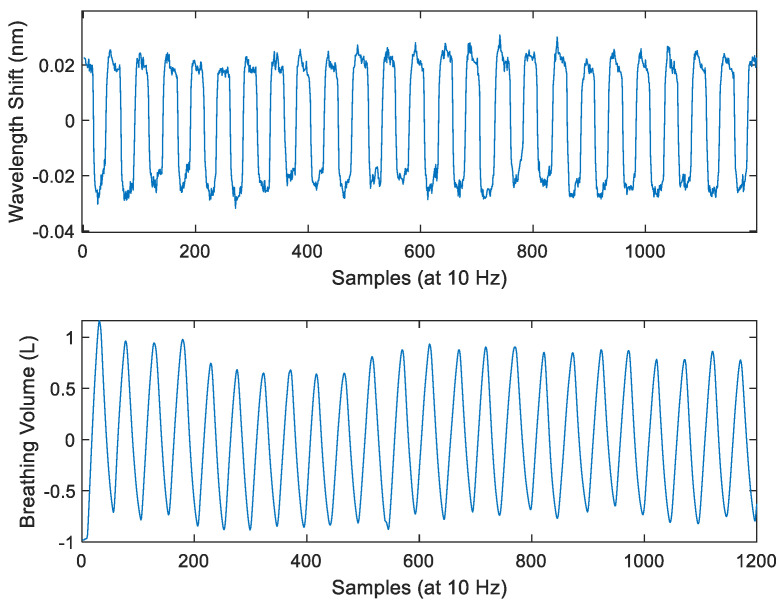
Examples of data from a volunteer (**top** panel = Mask System raw data, **bottom** panel = reference spirometer data).

**Figure 8:**
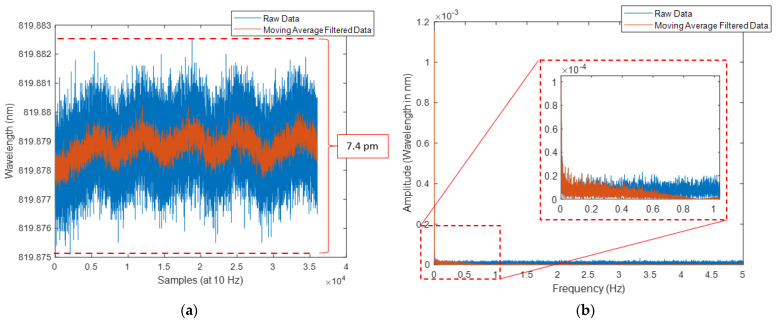
Mask System data when placed on benchtop at experiment venue; (**a**) time domain where the X-axis is labelled as data samples and acquisition rate; (**b**) frequency domain (note: sub-panel in (**b**) shows magnification of data at 0–1 Hz).

A typical example of raw volunteer studies data from the Mask System and reference spirometer is shown in Figure 7. It is observed that the Mask System data are the inverse of the reference spirometer data, which agrees with our expectation (Section 2.4.1). High-frequency noise (>0.4 Hz) is also present. This is consistent with Figure 8a, which shows the raw data from the Mask System when it is placed on a benchtop in the experiment venue with the same air conditioning setting as volunteer studies. Without the influence of breath-induced air temperature variation, there are high-frequency fluctuations with a similar magnitude to noise present in the volunteer study data. This indicates that the noise in the volunteer study signal is due to electrical and thermal noise in the mini-interrogator. Additionally, as the breath airflow slows at the peaks of inhalation and exhalation, further high-frequency fluctuations are likely contributed by increased air turbulence.

Figure 8b shows the benchtop data after filtering the raw benchtop data using a moving average filter. The magnitude of high-frequency fluctuation is greatly reduced (7.4 pm to 2.7 pm). Furthermore, the comparison between the Fast Fourier Transform (FFT) plot of the raw and filtered benchtop data showed that even though the high-frequency components of the signal are removed, the low-frequency ones remain largely untouched, including the 0.20 Hz to 0.33 Hz region, which is a normal human breathing frequency range.

**Figure 9:**
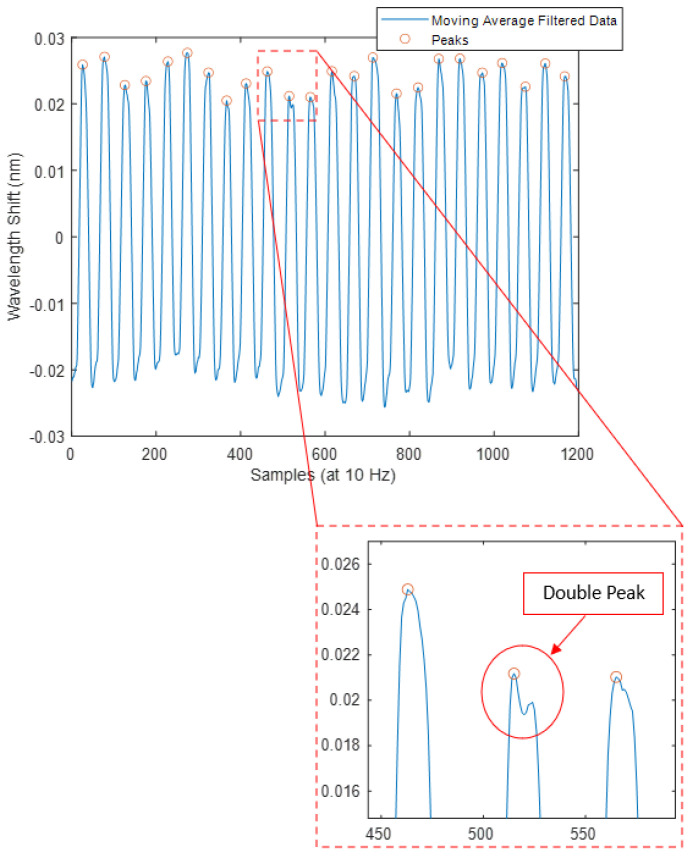
Inverted and filtered Mask System data peak detection (sub-panel shows magnification of data at the double peak).

In Figure 9, the Mask Data after filtering using the moving average filter and inversion is illustrated. The overall trend is retained, whilst the high-frequency fluctuations are mostly removed. Double peaks that remain in the filtered signal were not detected by findpeaks due to the minpeakprominence because their amplitudes were lower than 7.4 pm after filtering.

**Figure 10:**
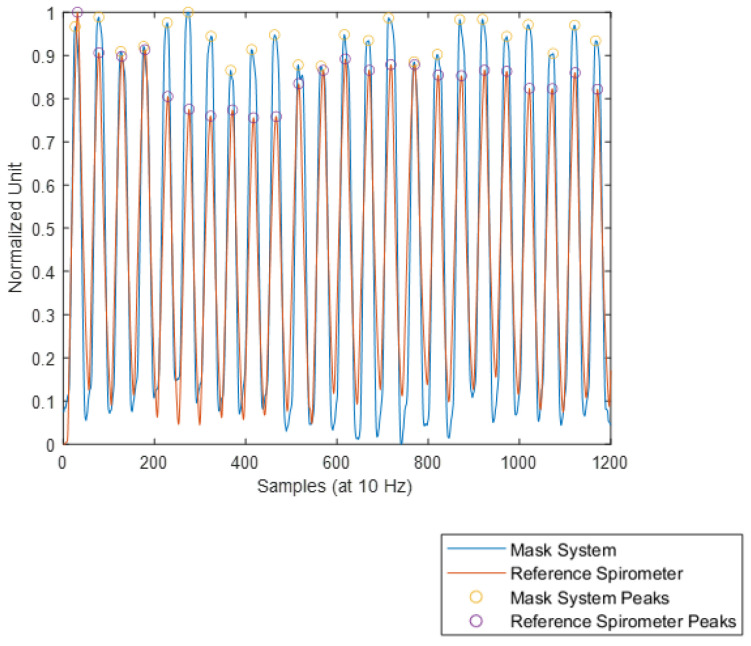
Example comparison between processed Mask System and reference spirometer normalised data and peak detection (note: the result of reference spirometer data manual breath count is shown as the peaks of the signal labelled as “Reference Spirometer Peaks”).

It can be seen from Figure 10 that the number of peaks counted from both plots of filtered-inverted Mask System data and reference spirometer data are identical (full set of normalised comparison plots of every volunteer given in Supplementary Materials). The resulting breath (peak) count from both the Mask System and reference spirometer for each volunteer is given in Table 3. Across every experiment repetition with every volunteer, the number of peaks counted by both the Mask System and the reference spirometer are identical.

## 4. Discussions

From Table 3, the Mask System and the reference spirometer have equal breath count through every volunteer trial, indicating 100% accuracy. This demonstrated improvements from our previous work [30] (65.4–88.1% mean accuracy vs. 100% accuracy). In the previous work, RR was acquired by performing a Fast Fourier Transform (FFT) on three data signals simultaneously obtained from two sensors featured in the same optical fibre placed within the EtCO_2_ (end-tidal CO_2_) mask. These include breath temperature-induced FBG wavelength shift, breath humidity-induced peak reflected light intensity variation, and breath humidity-induced variation in areas under the spectrum between 1529 and 1581 nm. The FFT was performed on the whole signal in every case, and the frequency corresponding to the greatest wavelength shift magnitude was recognised as RR. This method is prone to inaccuracy because the human respiration airflow pattern is not constant across every breath, even if the individual is breathing at a constant pre-determined RR. This indicates that the rate of corresponding temperature and humidity variation as measured by FBG also changes, which results in the frequency components of the breathing signal shifting from one that corresponds to the actual RR. However, the Mask System applies a peak detection algorithm directly onto the processed breath temperature-induced FBG signal. This better resembles the gold standard manual counting [47] and, thus, results in a more accurate RR measurement. Furthermore, the signals from peak reflected light intensity variation in our previous work were noisier than FBG wavelength shift. This is believed to be due to the position of the sensor within the EtCO_2_ mask, which is near the right cheek without touching the skin. This means that the sensor is subjected to more noise from air turbulence, compounded by the fact that the signal strength is reduced due to the FBG being further away from the mouth and nostrils. In contrast, the Mask System’s placement and orientation emphasise sensitivity to breath airflow in and out of the respirator mask (Section 2.1).

While we did not perform direct comparative tests with RR sensors from previous studies, our study involved volunteer experiments using tidal breathing, where both breathing rate and depth varied naturally, in contrast to studies that used pacing devices like metronomes [30]. This approach more closely mimics real-world clinical measurements of breathing rate. Direct comparison with published research on RR sensors is also challenging due to differences in performance evaluation protocols. For example, Bates et al. [9] measured data from a single volunteer over 6.9 h during sleep, Curone et al. [6] used exercises such as walking on slopes over 60 min, and Sinha et al. [22] focused on 60-s sessions with fixed breathing rates guided by a pacer. One study that employed a protocol similar to ours was Tavares et al. [22], which reported exact matches between the breathing rates measured by their system and a reference during tidal breathing (over 90 s in three intervals with 30 s of apnea in between). We observed comparable performance with our Mask System. However, as shown in Table 1 and Table 2, the Mask System offers key advantages over Tavares et al.’s approach and other studies: its portability and its resistance to noise caused by respiration-irrelevant torso movements. This makes it more suited for use during dynamic activities, such as manual labour, where torso movement can interfere with sensor accuracy. We believe these advantages highlight the Mask System’s potential for practical, real-world applications.

Although the results were promising, the volunteer study was performed in a controlled environment, and the Mask System does not feature air filters, which are typically present in industrial protective respirator masks. Therefore, it is proposed that additional investigations are conducted before work-simulated and real-life testing (different breathing rates in various mining and factory environments whilst performing various physical activities). These include investigations of (1) the effects of filters and different mask designs and (2) FBG pressure sensors.

(1) Effect of mask structures and filters—during mining and factory work, workers are expected to wear industrial-grade respirator masks fitted with filters (such as P100, Honeywell International, Charlotte, NC, USA) to protect them from airborne harmful substances. They also typically have separate valves for inhalation and exhalation (for example, PD-101 Full-Face Industrial Air Purifying Respirator, Parcil Safety, Evansville, IN, USA). In some cases, a dedicated electric-powered portable air purifying unit is also included to pump filtered air into the mask (for example, Versaflo TR-800, 3M, Maplewood, MN, USA). These features can introduce different air movements and turbulence patterns than the respirator mask currently used in the Mask System. Thus, this investigation will include studying the effect of filter addition to the respirator mask airway, different airway structures, and different FBG placements in these structures to find the placement protocol that produces the greatest ratio of FBG sensitivity to breath-induced temperature variation and the noise from air turbulence.
(2) FBG pressure sensor—in the environment where the ambient temperature is high or varies greatly (such as during underground mining where workers can face temperatures up to 37 °C [48]), the temperature of the exhaled breath and surrounding air can be comparable, making it difficult to identify the peak in the signal with only the findpeaks function. A solution to this is to incorporate an FBG pressure sensor into the Mask System to detect pressure changes due to inhalation and exhalation [49]. If ambient air temperature and breath temperature are significantly different, then the temperature sensor is advantageous to the pressure sensor in detecting inhalation and exhalation during quiet breathing, where the pressure drop is low due to small airflow [50]. On the other hand, in the case of minor temperature differences (causing less wavelength shift than the Mask System’s noise shown in Figure 8a, pressure measurement data can be used to assist the breath detection algorithm. Thus, research to design the FBG air pressure sensor and placement within the mask for accurate breath detection is required.

Since the interrogator (Fispec FBG X100 FiSens GmbH, Braunschweig, Germany) can support multiple FBGs simultaneously (up to 30), a wide range of FBG-based sensor systems can be integrated in addition to the temperature and pressure sensors. For example, the Mask System can be integrated with a heart rate (HR) monitoring strap (1 FBG) [32] and body temperature monitoring system (1 FBG) [51] to detect physical stress, heat stress, and fatigue, which will further contribute to the safety of workers in harsh environments [52,53].

Table 4 compares the Mask System’s mini-interrogator and other methods, both commercial and research. With regards to commercial methods, the main advantage over the proposed system is the number of supportable FBGs. However, commercial interrogators are bulkier and heavier, making them unsuitable for applications involving portable, real-time measurements during daily/occupational tasks. For novel methods, some reported full integration into a textile, i.e., each component of the interrogation system being attached to a shirt distributing the weight of the apparatus along the surface area, which favors convenience and comfort. However, since mine and factory workers may have to carry heavy tools or equipment on their body, such as an over-the-shoulder tool belt or an air purifying unit in industrial-grade protective mask, the components and wiring can be damaged. Furthermore, in these novel methods, the number of FBG supported is also lower, with some having a lower wavelength range, hence limiting the potential for multiple sensor integration.

## 5. Conclusions

In summary, an RR monitoring device based on incorporating FBG sensors into a respirator mask has been developed. A performance evaluation experiment with 10 healthy volunteers showed that the device could detect inhalation and exhalation coinciding with that of the reference spirometer, where both suggested the same number of breaths across the period of 120 s. Based on the results, the Mask System demonstrated improved RR from our previous work. The application of the moving average filter and peak detection algorithm directly onto the FBG signal in the time domain showed that the individual breath can be distinguished from the influence of air turbulence and accurately detected.

The employed mini-interrogator also demonstrated its ability to perform continuous measurements error-free across 10 volunteer experiments, which represents reliability in miniature FBG interrogation technology. This suggests the potential and opportunity to develop FBG technology into portable devices that can display and transfer medical data wirelessly and in real time. Overall, the Mask System showed great potential for future development as a reliable means of RR monitoring for workers. However, the system still lacks features such as a filter and dedicated valve for inhalation and exhalation typically found in industrial-grade protective masks. Additionally, the volunteer study was performed in a closed and controlled laboratory environment. Therefore, research proposals were discussed to address these and further develop the Mask System for deployment in harsh environments.

## Figures and Tables

**Table 1:** Chronological review of RR monitoring device operation principle and performance/error (n = no. of volunteer/subject, MPE = mean percentage error, SD = standard deviation, COV = coefficient of variation, BA = Bland–Altman, LOA = limits of agreement, RMSE = root mean square error, CC = correlation coefficient, BPM = breaths per minute, BREA = breathing rate estimation accuracy = [1—normalized mean absolute error] × 100%).

Author, Reference, and Year	Operation Principle	n	Portable	Performance/Error	Limitations
Bates et al., [9], 2010	Accelerometer (chest wall angular displacement)	1	No	RMSE: 0.38 BPM (3 BPM peak error)	Prone to respiration-irrelevant motion artefacts
Curone et al., [6], 2012	Piezoelectric sensor strap (voltage variation due to respiration-induced stress)	6	No	BA: bias range = −5 to 0 BPM, max upper LOA = 11, min lower LOA = −17	
Fekr et al., [10], 2018	Accelerometer (chest wall 3D movement)	8	No	Mean CC: 0.85 BPM, SD not given	
Massaroni et al., [16], 2018	RGB video analysis	12	No	BA: −0.03 BPM bias (−3.51 to 3.46 BPM LOA)	Prone to interference due to camera obstructions and respiration-irrelevant motion artefacts
Aqueveque et al., [11], 2020	Thoracic impedance plethysmography (four electrodes, two on each hemithorax)	15	No	BA: right thorax (−0.18 BPM bias, −2.96 to 2.6 LOA), left thorax (−0.52 BPM bias, −3.08 to 2.07 LOA)	Prone to inaccuracies due to motion artefacts and breathing movement pattern variation
Kwon et al., [12], 2020	Thermography imaging (respiration-induced temperature variation near nostril)	101	No	BA: −0.15 BPM bias (2.65 to −2.92 LOA)	Prone to interference due to camera obstructions and other movements
Hayward et al., [13], 2022	Capaciflector (chest wall motion)	61	No	BA: static (bias range = 0.95 to 0.85 BPM, min lower LOA = −2.30, max upper LOA = 3.90); exercise (bias range = 0.31 to 0.32 BPM, min lower LOA = −3.51, max upper LOA = 3.36)	Prone to cross-talks due to moisture buildup in the sensing element due to skin humidity and sweating
Dawood et al., [14], 2022	Thermistor mask (respiration-induced temperature variation near nostril)	5	Yes	MPE: 5.6% (SD not reported)	Prone to EM-induced self-heating
Bhongade et al., [17], 2023	Inertial measurement unit (three units placed on chest, abdomen, and lateral left rib)	3	Yes	BREA: 97.50 ± 1.17% (static seated); 96.84 ± 1.76% (seated working); and 95.17 ± 2.66% (static supine)	Prone to respiration-irrelevant motion artefacts

**Table 2:** Chronological review of fibre optic respiration sensor operation principle, respiration parameter measured, calibration methodology, and performance/error (n = no. of volunteer/subject, MPE = mean percentage error, SD = standard deviation, BA = Bland–Altman, LOA = limits of agreement, MPA = mean percentage accuracy, MAPE = mean absolute percentage error.

Author, Reference, and Year	Operation Principle	n	Portable	Performance/Error	Limitations
Vegfors et al., [34], 1993	Boundary reflection	10	No	MPE: spontaneous rate (0.1%, 1.91% SD), fixed rate (0%, 0.67% SD)	Prone to respiration-irrelevant motion artefacts
Yoo et al., [26], 2011	Polycrystalline optical fibre, thermochromic microcapsule pigment (respiration-induced temperature variation near nostril)	1	No	No comparison with reference devices reported	Prone to respiration-irrelevant motion artefacts, discomfort due to placement requirements (directly on skin between nose and upper lip via adhesive)
Dziuda et al., [27], 2013	FBG-embedded plexiglass bed (torso motion)	3	No	BA: 0.00 BPM bias (–0.42 to 0.43 LOA)	Applicable only for static supine position
Elsarnagawy, [28], 2015	FBG-embedded strap (horizontal chest strain)	10	No	Exact match with manual counting	Prone to respiration-irrelevant motion artefacts, placement-induced discomfort (directly over mammilla)
Presti et al., [29], 2018	FBG array (body strain)	8	No	PE: 0.22% mean (0.12% SD)	Prone to respiration-irrelevant motion artefacts
Sinha et al., [30], 2021	FBG (respiration-induced temperature variation near nostril)	18	No	MPA: 88.1% (2.9% SD)	Interpretation algorithm undermines breathing frequency variation during measurements
Di Tocco et al., [31], 2021	FBG (horizontal chest and abdomen strain)	10	No	MAPE: 2.34% (1.88% SD)	Prone to respiration-irrelevant motion artefacts
Tavares et al., [32], 2022	FBG (horizontal chest strain)	3	No	Exact match with reference RR	
Roudjane et al., [35], 2018	Hollow-core silica-based fibre array bending (horizontal chest strain)	4	Yes	No comparison with reference devices reported	Prone to respiration-irrelevant motion artefacts

**Table 3:** No. of breaths counted from Mask System and reference spirometer data throughout the duration of each repetition of tidal breathing experiment (120 s).

Volunteer ID	Mask System Breath Count (First, Second, Third, Fourth, Fifth Experiment Repetitions)	Reference Spirometer Breath Count (First, Second, Third, Fourth, Fifth Experiment Repetitions)
18131	24, 25, 21, 23, 23	24, 25, 21, 23, 23
20609	37, 38, 39, 38, 38	37, 38, 39, 38, 38
56464	37, 37, 35, 34, 31	37, 37, 35, 34, 31
57384	33, 33, 28, 28, 27	33, 33, 28, 28, 27
63350	19, 23, 22, 23, 20	19, 23, 22, 23, 20
65714	31, 31, 29, 31, 31	31, 31, 29, 31, 31
66409	20, 24, 19, 20, 22	20, 24, 19, 20, 22
77711	20, 20, 20, 20, 19	20, 20, 20, 20, 19
89277	36, 33, 40, 37, 41	36, 33, 40, 37, 41
95543	25, 33, 34, 35, 34	25, 33, 34, 35, 34

**Table 4:** Comparison between proposed Mask System and other methods of interrogation, both commercial and novel (NR = not reported; * = individual components of apparatus reported to be placed in a single layer directly onto a shirt).

System		No. of Channels	Max No. of FBG per Channel	Sampling Rate (hz)	Wavelength Range (nm)	Resolution (nm)	Dimension (mm × mm × mm)	Weight (g)	Power Consumption (W)	Battery-Powered
Mask System		1	30	10	808–880	0.1	155 × 86 × 65	374	1.2	Yes
Commercial	SmartScan (Smartfibres, Bracknell, UK)	4	16	2500	1528–1568	0.1	140 × 115 × 85	900	8.5	No
	LCM-2708 (Fibrestrike, Twinsburg, OH, USA)	8	8	19,200	1515–1585	1	170 × 99 × 361	4536	NR	Yes
	FSI S16PC (Femto Sensing, Atlanta, GA, USA)	16	30	1	1510–1590	1	235 × 202 × 120	3500	18	No
	Hyperion Si255 (Luna Innovations, Roanoke, VA, USA)	16	*	5000	1460–1620	NR	307 × 274 × 69	4900	40	No
Novel	Carmo et. al. (2012) [54]	1	16	36	1529–1561	1	NR *	250	1	NR
	Diaz et. al. (2019) [55]	1	*	213	1522–1567	3.82	145 × 85 × 45	300	3	Yes
	Tian et. al. (2021) [56]	4	*	4000	1510–1595	0.1	NR	NR	6	NR
	Li et. al. (2012) [57]	1	3	500	1540–1560	0.08	NR *	10	NR	Yes

## Data Availability

The data presented in this study are available upon request from the corresponding author.

## Supplementary Materials

The following supporting information can be downloaded at: https://www.mdpi.com/article/10.3390/s24237476/s1, Figure S1: Volunteer ID. 18131 Mask System vs. reference spirometer data (top to bottom plots represent experiment repetitions 1–5, respectively); Figure S2: Volunteer ID. 20609 Mask System vs. reference spirometer data (top to bottom plots represent experiment repetitions 1–5, respectively); Figure S3: Volunteer ID. 56464 Mask System vs. reference spirometer data (top to bottom plots represent experiment repetitions 1–5, respectively); Figure S4: Volunteer ID. 57384 Mask System vs. reference spirometer data (top to bottom plots represent experiment repetitions 1–5, respectively); Figure S5: Volunteer ID. 63350 Mask System vs. reference spirometer data (top to bottom plots represent experiment repetitions 1–5, respectively); Figure S6: Volunteer ID. 65714 Mask System vs. reference spirometer data (top to bottom plots represent experiment repetitions 1–5, respectively); Figure S7: Volunteer ID. 66409 Mask System vs. reference spirometer data (top to bottom plots represent experiment repetitions 1–5, respectively); Figure S8: Volunteer ID. 77711 Mask System vs. reference spirometer data (top to bottom plots represent experiment repetitions 1–5, respectively); Figure S9: Volunteer ID. 89277 Mask System vs. reference spirometer data (top to bottom plots represent experiment repetitions 1–5, respectively); Figure S10: Volunteer ID. 95543 Mask System vs. reference spirometer data (top to bottom plots represent experiment repetitions 1–5, respectively).
