# Supplementary material for: Respiratory Rate Monitoring via a Fibre Bragg Grating-Embedded Respirator Mask with a Wearable Miniature Interrogator
Source: Sensors (Basel). 2024 Nov 23;24(23):7476. doi: 10.3390/s24237476 (PMC11644672; doi:10.3390/s24237476)
Supplement: Supplementary file 1 [file sensors-24-07476-s001.zip › sensors-3310564-supplementary.pdf]

<u>Supplement A</u>: Mask System vs Reference Spirometer

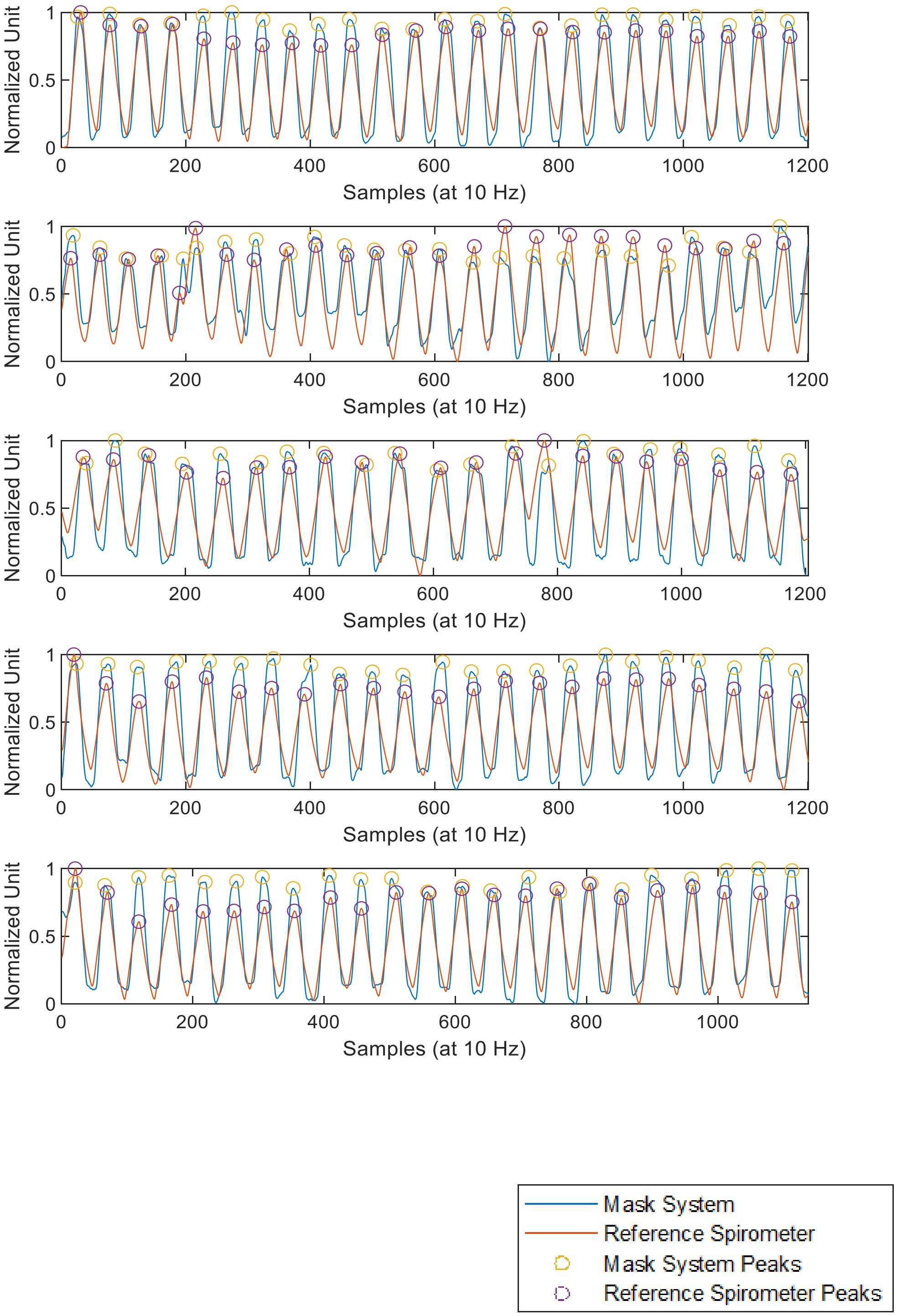

Figure S1: *Volunteer 18131 Mask System vs reference spirometer data (top to bottom plot represent experiment repetition 1 – 5, respectively)*

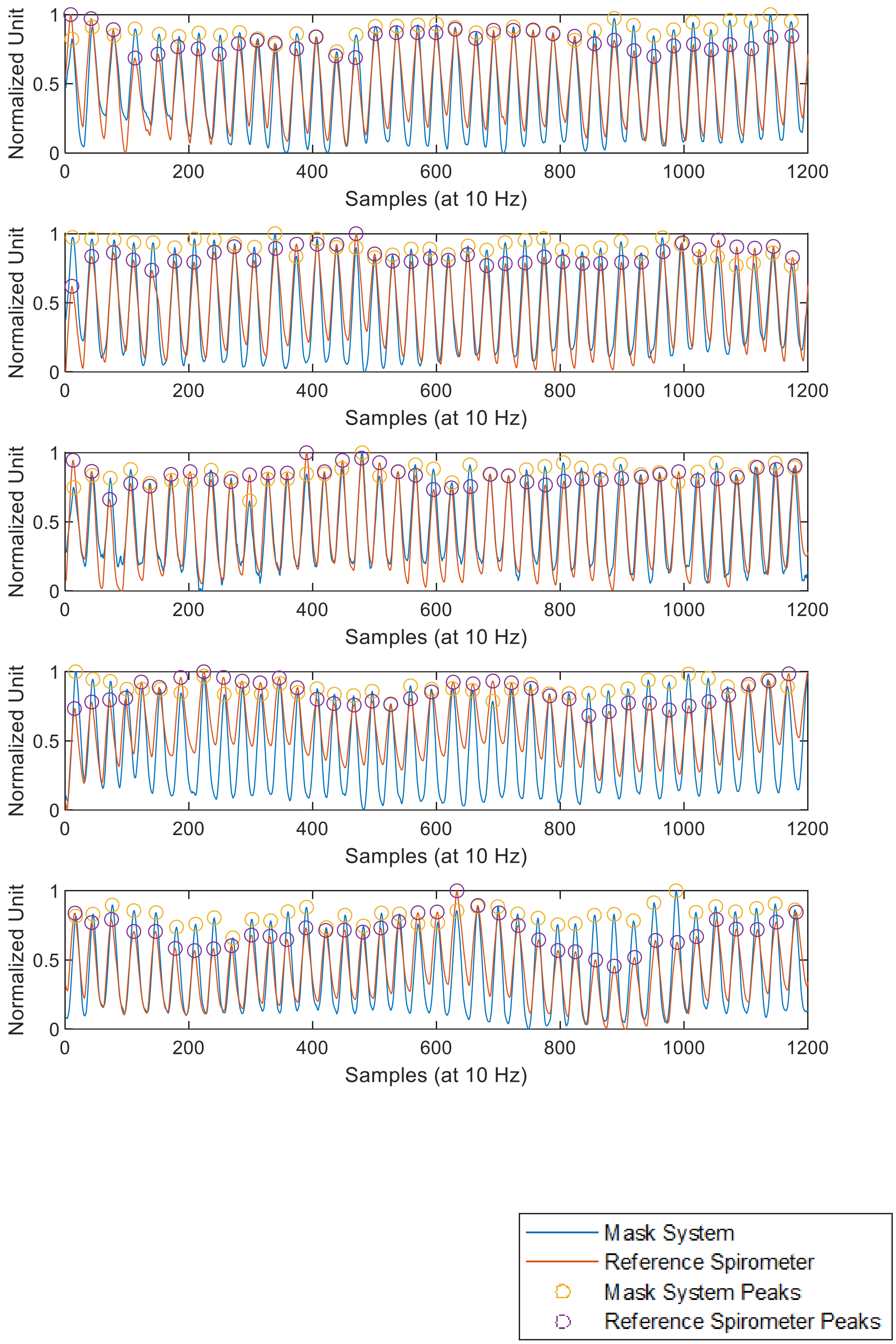

Figure S2: *Volunteer 20609 Mask System vs reference spirometer data (top to bottom plot represent experiment repetition 1 – 5, respectively)*

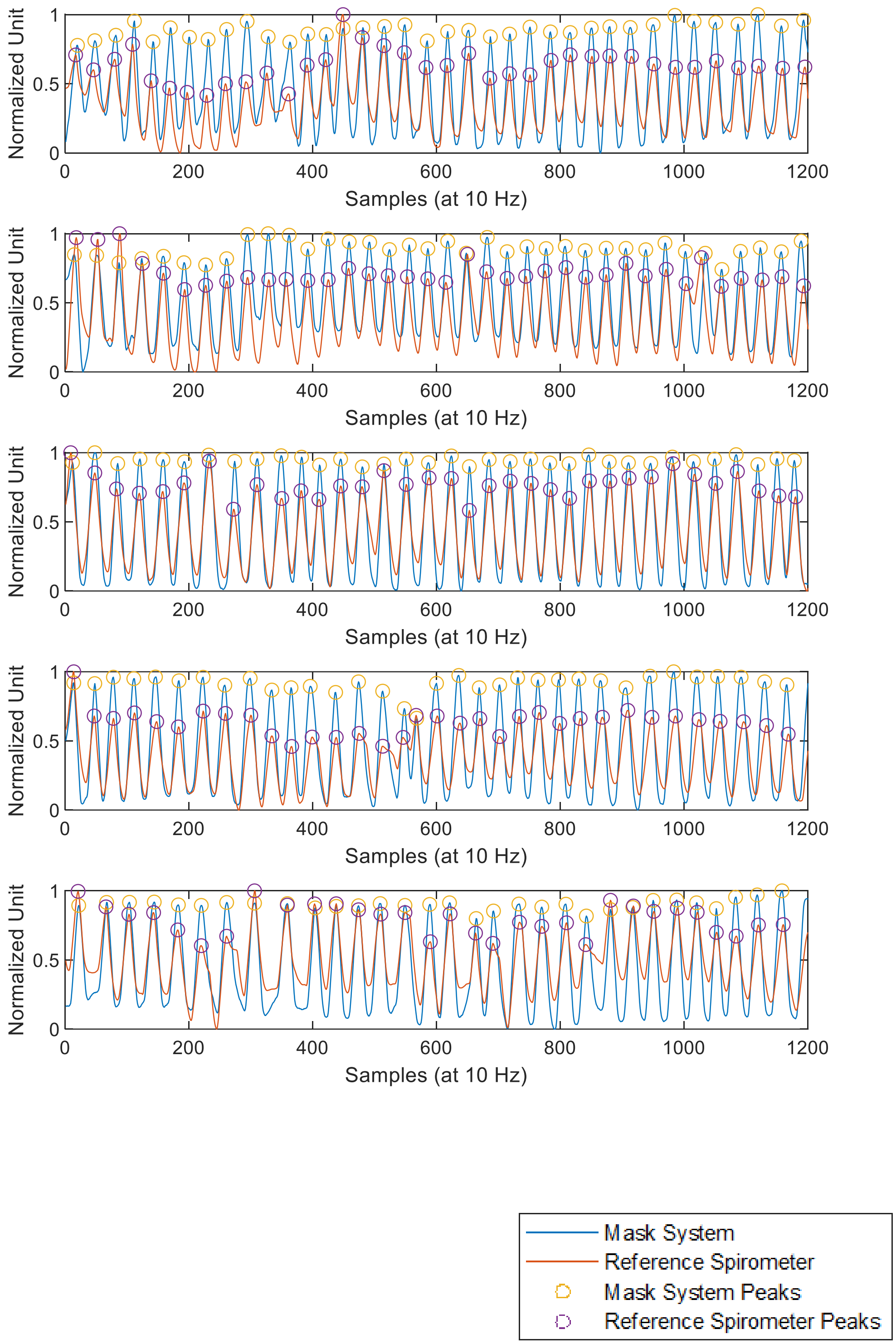

Figure S3: *Volunteer 56464 Mask System vs reference spirometer data (top to bottom plot represent experiment repetition 1 – 5, respectively)*

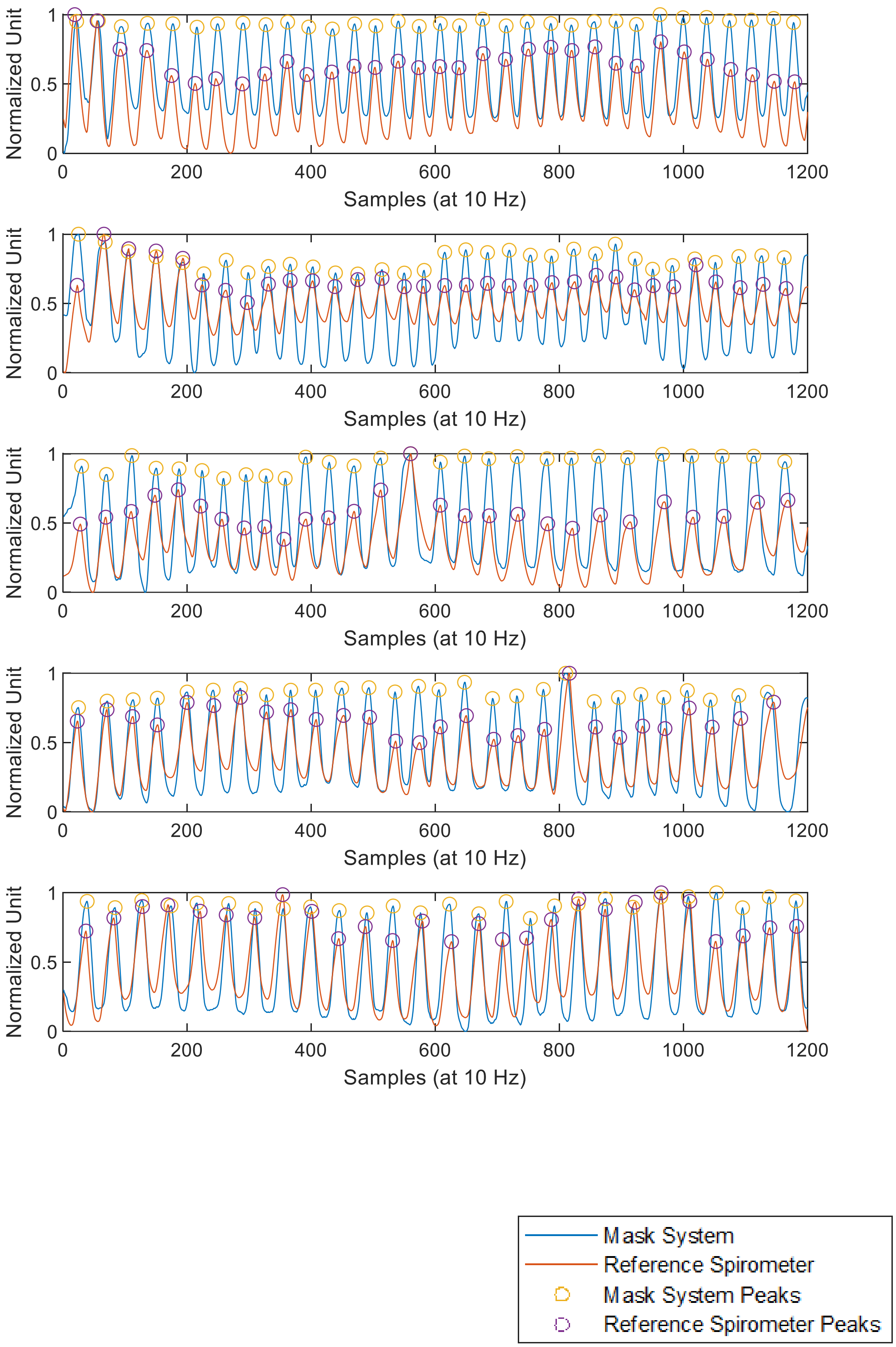

Figure S4: *Volunteer 57384 Mask System vs reference spirometer data (top to bottom plot represent experiment repetition 1 – 5, respectively)*

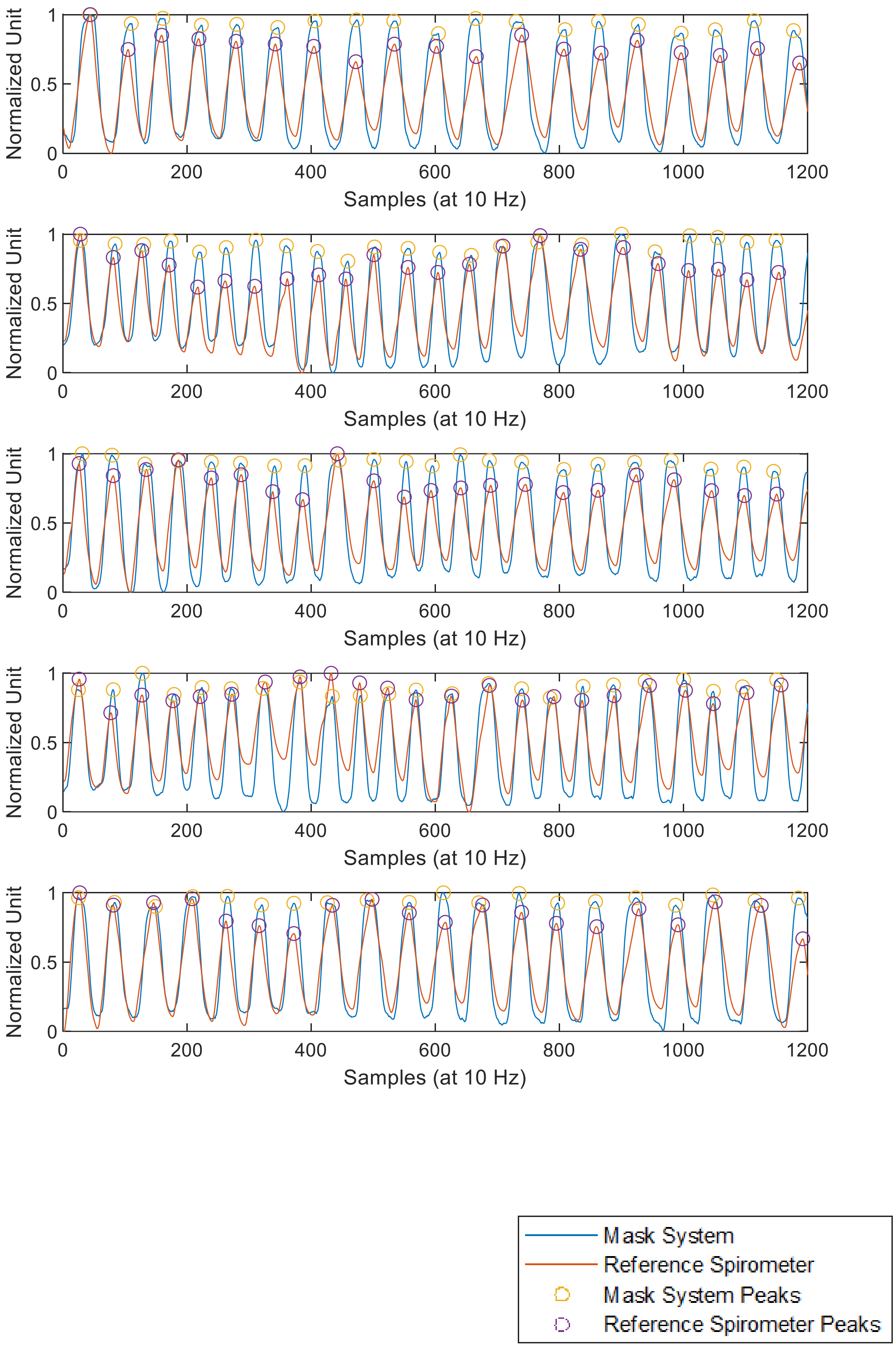

Figure S5: *Volunteer 63350 Mask System vs reference spirometer data (top to bottom plot represent experiment repetition 1 – 5, respectively)*

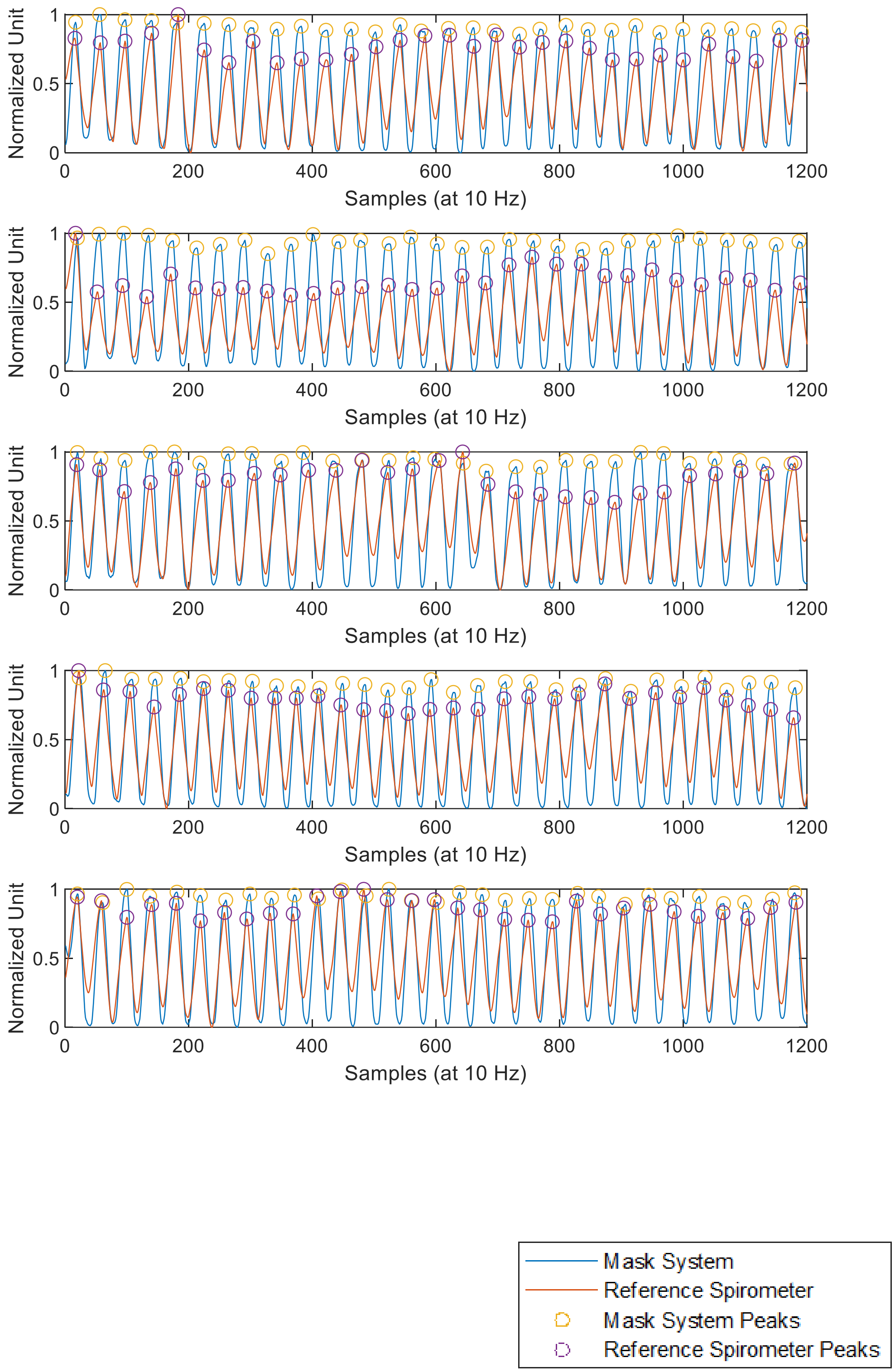

Figure S6: *Volunteer 65714 Mask System vs reference spirometer data (top to bottom plot represent experiment repetition 1 – 5, respectively)*

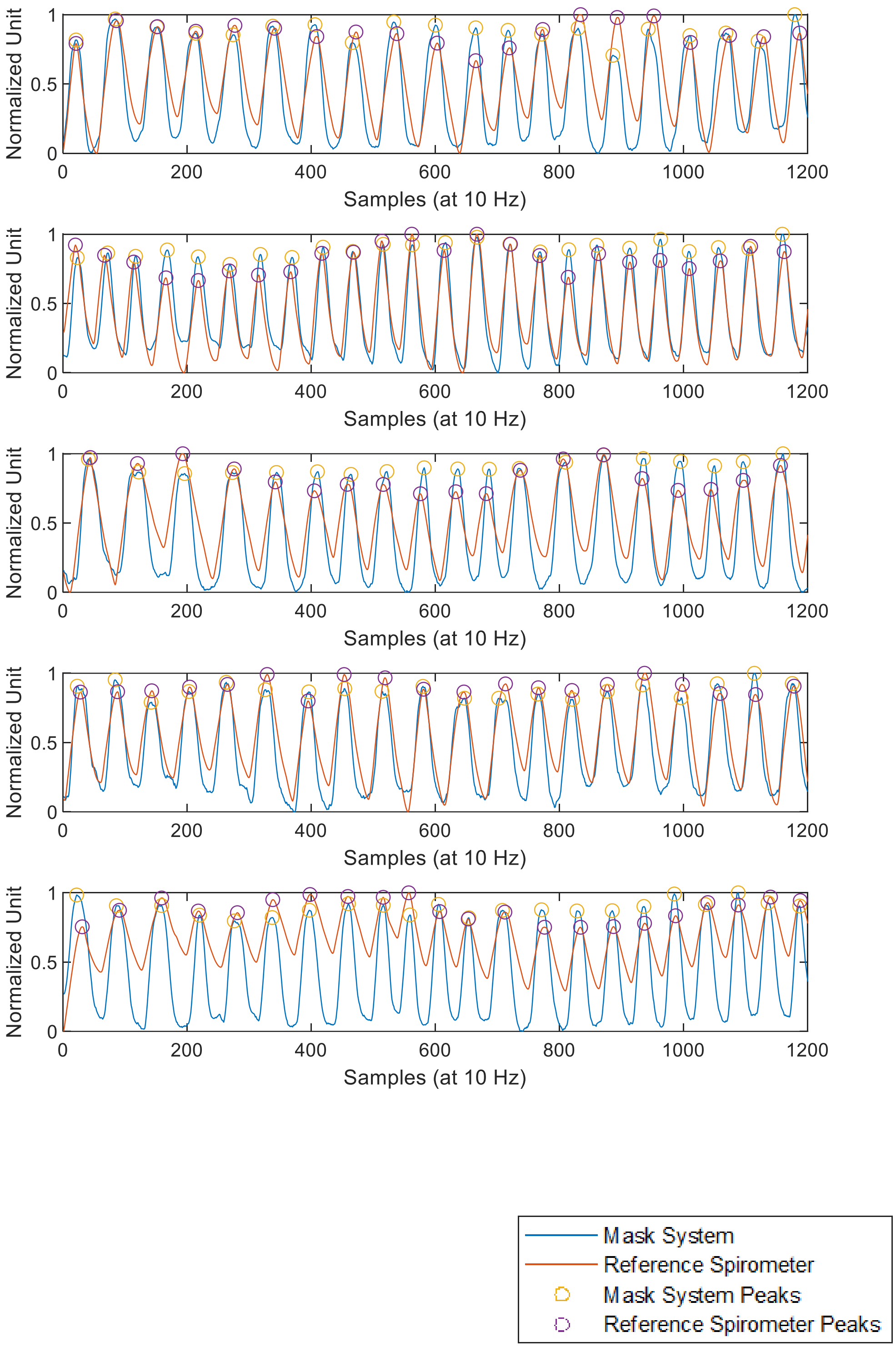

Figure S7: *Volunteer 66409 Mask System vs reference spirometer data (top to bottom plot represent experiment repetition 1 – 5, respectively)*

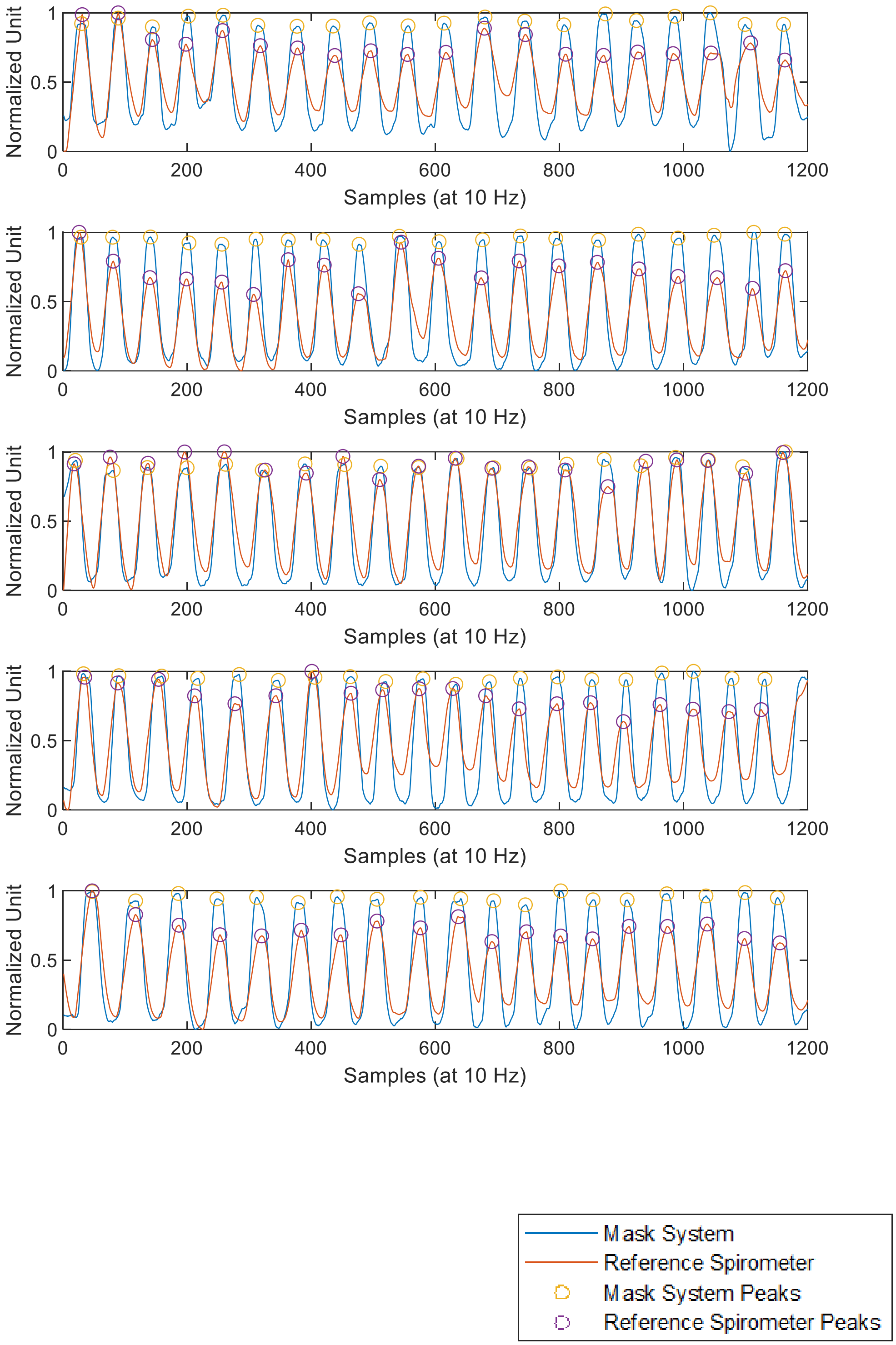

Figure S8: *Volunteer 77711 Mask System vs reference spirometer data (top to bottom plot represent experiment repetition 1 – 5, respectively)*

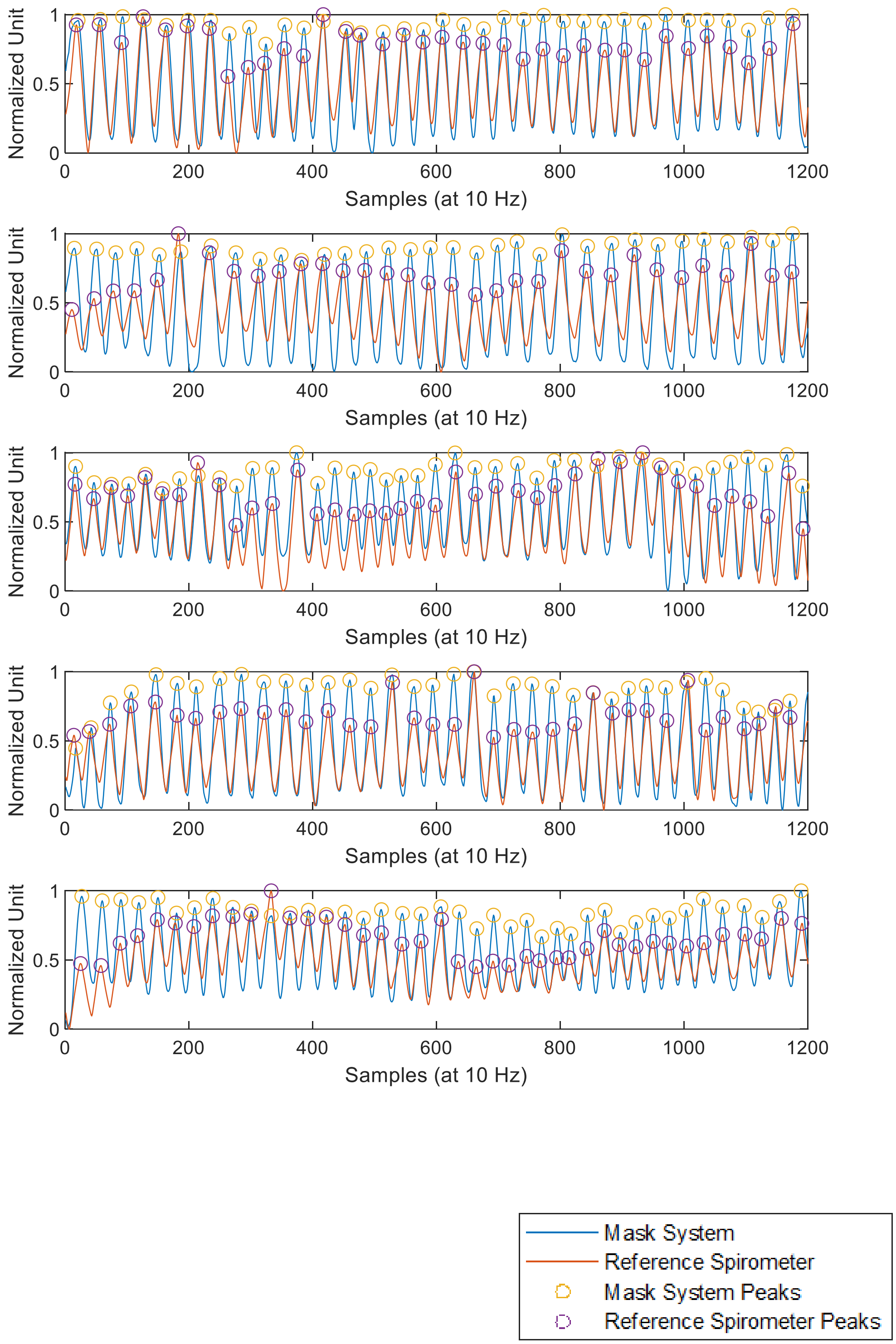

Figure S9: *Volunteer 89277 Mask System vs reference spirometer data (top to bottom plot represent experiment repetition 1 – 5, respectively)*

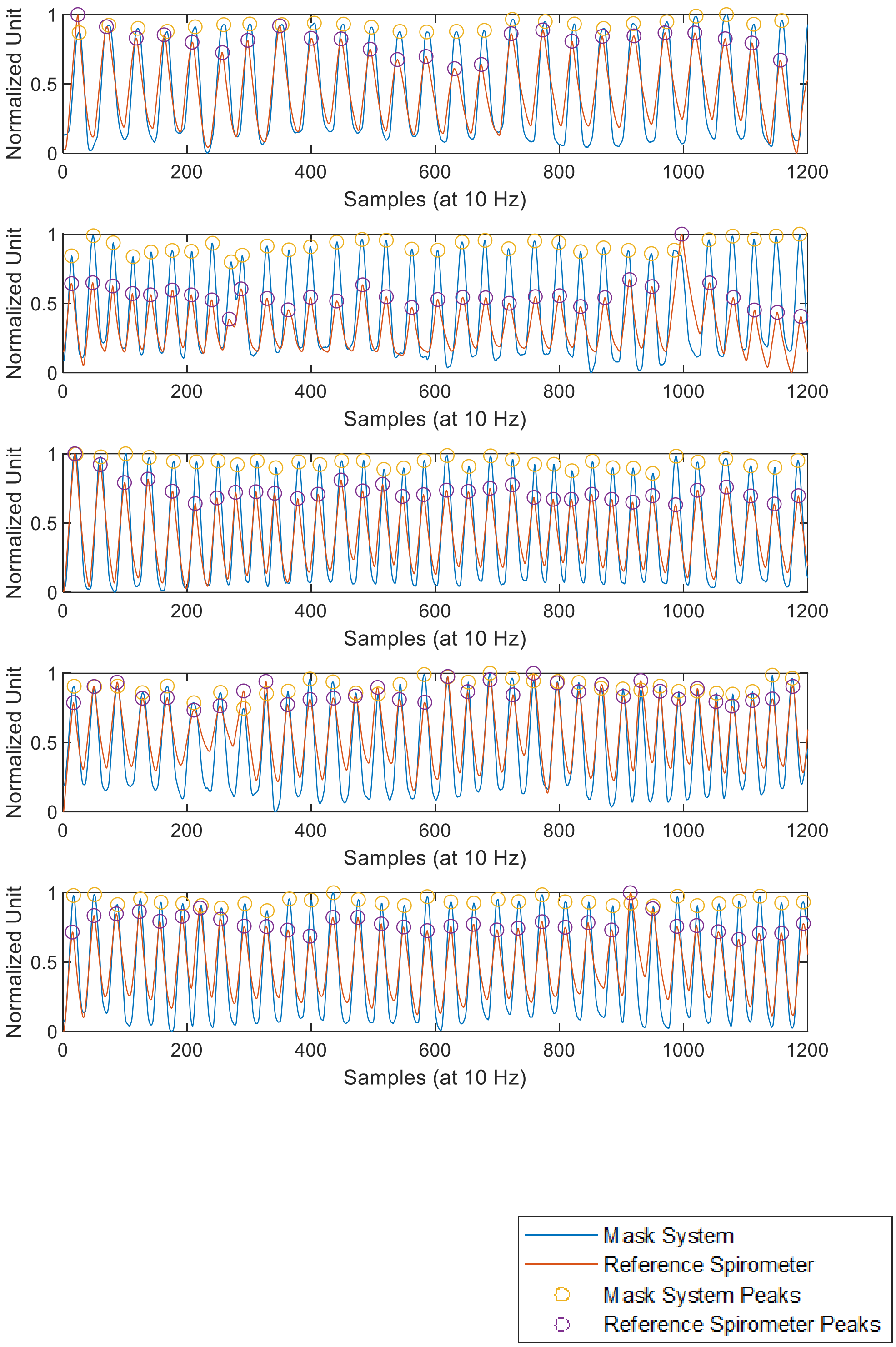

Figure S10: *Volunteer 95543 Mask System vs reference spirometer data (top to bottom plot represent experiment repetition 1 – 5, respectively)*
